# Tax structure, government debt, and the relative power of local education supply

**DOI:** 10.1371/journal.pone.0301985

**Published:** 2024-06-11

**Authors:** Mengqin Li

**Affiliations:** Economics and Management School of Wuhan University, Wuhan University, Wuhan, Hubei, China; University of Murcia: Universidad de Murcia, SPAIN

## Abstract

Policymakers are increasingly focusing on structural adjustment and efficiency to cope with the pressures that the economic downturn has placed on local finances. Accordingly, the Chinese government should shift from using standard passive investments to high-quality active investments for its social guarantees, such as education. Based on panel data of 274 cities from 2010 to 2019, this study conducted the first examination of the impact of tax structure and government debt on the relative power of the local education supply (LES) in China. The study found that, first, in general, increases in the tax structure—represented by the proportion of personal income tax to budgetary revenue strengthen the relative power of LES, which is more sensitive in the southern region with a more developed market economy system. And the impact of government debt—represented by the urban investment debt ratio on the relative power of LES is initially negative and then positive. Second, the study revealed that the tax structure can stimulate the relative power of LES through the intermediary channel of an increase in the urban consumption rate; however, the mechanism of promoting the relative power of LES by encouraging localities to attract more floating populations is not obvious. Third, excessive investment in local governance adjusts the positive effect of local debt on the relative power of LES. Therefore, the government should pay attention to the promotion of personal income tax status, standardize their debt risk management, improve the efficiency of governance, and emphasize the pull of urban consumption, so as to enhance the ability to support livelihood and fully mobilize initiatives for local education development.

## 1. Introduction

Balanced education is an important part of the UN’s sustainable development framework and a fundamental solution to inequality of opportunity. Table 1 shows a clear gap between China’s public education expenditures and the global average growth rate. China is a developing country with a socialist political and economic system and the world’s largest population. Thus, it is of great theoretical and practical significance to study and solve the problem of education balance in China to realize the balanced and sustainable development of global education. As of 2020, China has eliminated absolute poverty and shortages. Today, China is working to ensure that its society is moderately prosperous in all respects. At this stage, China’s work to supply livelihood services, such as education, should focus on balancing regional disparities and transitioning to active competitive supply (rather than passive index supply). Hence, this work requires enhancing the relative power of the local education supply (LES) in various regions.

**Table 1:** Public education expenditures for the world, the US, Britain, China, Russia, and India.

Country Name	Indicator Name	2010	2020	Growth Rate (%)
World	The proportion of public expenditure on education to GDP	4.2	4.33	3.18
The United States	The proportion of public expenditure on education to GDP	6.72	6.05	-9.97
Britain	The proportion of public expenditure on education to GDP	5.67	5.53	-2.44
China	The proportion of public expenditure on education to GDP	3.75	3.57	-4.8
Russian federation	The proportion of public expenditure on education to GDP	3.88	3.73	-3.88
India	The proportion of public expenditure on education to GDP	3.38	4.47	32.46

Source: World Bank, World Bank Database

This study considered the relative power of LES, highlighting its proactive characteristics by balancing its inter-regional gaps. Previous studies used the proportion or coefficient of variation of education expenditure to represent education equity. Thus, they have only considered the absolute amount or gap in local education expenditure; this approach fails to fully reflect the active competitive supply of inter-regional education expenditure, which plays a key role in the balanced development of essential public services across China.

In China, local governments are the leading suppliers of public goods, such as education, and local government finance is one of the largest suppliers and factors of local education. Existing literature mainly studies the supply of livelihood security, such as local education, in light of fiscal decentralization [1–3], fiscal competition [4, 5], transfers [6–8], and fiscal pressures [9, 10]. Moreover, it pays little attention to budgetary structures—an important part of the financial system. An in-depth analysis of financial structure can yield more systematic and comprehensive financial theory that is applicable to LES.

Meanwhile, as the largest developing country, China is dominated by indirect rather than direct taxes and many local urban investment bonds. Analyzing the impact mechanism of this special fiscal structure on LES can yield an international comparison and reference. Additionally, given increasing fiscal pressure, government debt risk, and the fiscal expansion of productive expenditure, China must urgently explore a new channel for adjusting tax and debt structures for education and other livelihood expenditures to reduce imbalances in the supply of public goods and sustainably develop local education.

In addressing these concerns, this study makes three contributions. First, to support equal and high-quality public support in China’s new era, it proposes the concept of the relative power of LES and constructs the relative power index of LES. Second, based on the economic background of China’s indirect tax and many urban investment bonds, this study clarifies the influence of the local tax structure and government debt on the relative power of LES from theoretical and empirical perspectives, providing a theoretical and practical sample for the balanced and sustainable development of global education. Finally, the study uncovers the intermediary and adjustment mechanisms of the local tax structure and government debt affecting the relative power of the LES and provides policy implications.

## 2. Literature review

Existing scholarship on the influencing factors of LES primarily focuses on topics such as public choice, fiscal decentralization, and political centralization theories [11–14] and uses demand expression, fiscal, and political centralization characteristics, and macroeconomic systems as explanatory factors. Previous studies have shown that, although China’s fiscal decentralization and competition have increased local economic and budgetary revenues and can increase productive government investment, they are not conducive to the total supply of public goods for livelihood [4, 15]. To find another way to improve the supply of public goods for livelihood, scholars have focused on central government transfer payments. Li et al. [7] and Fu et al. [6] believe that a certain amount of special transfers play a positive role in the supply of public goods for livelihood. However, general transfer payments (which account for most transfer payments) stimulate local investment in production and objectively serve as a "soft budget payment" for local governments, which makes the regional government passively dependent on the transfer payment for the supply of public goods for livelihood. Meanwhile, scholars have proposed a unique Chinese-style promotion tournament model and argued that the political promotion model with GDP as the assessment target compels local governments to affirm productive public goods and dislike those related to livelihood. However, after the central government added livelihood evaluations to the assessment system, local governments significantly increased their livelihood expenditures [16]. From the perspective of factional politics, Li et al. [17] argued that officials with weaker political factions would strengthen the supply of public goods for livelihood, while those with more robust politics would not.

Fiscal pressure, tax sharing between superior and subordinate governments, and fiscal structure have become popular factors in the supply of public goods for livelihood with the recent decline in economic growth. Many studies suggest that fiscal pressure is the primary reason for the reduction in the bias of local government expenditures on livelihood [10, 18]. For example, Wu and Zhou [9] argue that local governments will adopt a "keep and compress" expenditure strategy as financial pressure increases, showing a tendency to produce and construction expenditures and reduce livelihood expenditures. In terms of tax sharing between superior and subordinate governments, Chu and Zhang [19] believe that the increase in local tax sharing has a positive but not significant impact on local livelihood expenditures. Xie and Zhang [20] believe that different types of public goods have different effects on the growth of tax bases in various industries; moreover, tax sharing will promote the allocation of public goods enhancing the tax base, while changes in value-added tax (VAT) sharing are insufficient to stimulate local governments’ allocation of cultural and other livelihood public goods. In terms of the tax structure, taxation most directly impacts national fiscal revenue and spending. In Zhang et al.’s [21] endogenous growth model, financing public service expenditure through income tax revenue and funding public capital through consumption tax revenue can effectively reduce tax system distortions. Li and Lin [22] predict that the introduction of property tax, as a new hot topic in China’s tax system reform, will increase local investment in material and human capital, improving the situation for future generations. Pang et al. [23] empirically conclude that the regional income tax growth rate significantly increases livelihood expenditures. Guo et al. [24] empirically studied prosperity and showed that the proportion of direct tax is significantly positively correlated with the level of shared prosperity; by contrast, the proportion of indirect tax demonstrates the opposite relationship—ultimately, they concluded that common prosperity depends on the gradual increase in direct taxes, especially personal income tax (PIT) and property taxes. In sum, existing literature mostly focuses on the impact of fiscal decentralization, pressure, and competition on livelihood expenditures [18, 25]. Few studies consider the impact of tax revenue structure, and those that do have used theoretical modeling [21] or empirical analyses that do not fully explore their impact mechanism [23, 24, 26].

As a key component of local livelihood expenditures, LES has grown significantly in the past few decades. However, local governments are still more attentive to productive investments, resulting in significantly imbalanced and inadequate LES across regions. Scholars have already considered the absolute expenditure of local education or the absolute disparity in education between regions, but the mainly unbalanced and inadequate contradiction at this stage is reflected more greatly in the relative differences. Stimulating the power of the local initiative supply can solve this contradiction. Therefore, this study explored the relative power of LES and the relative supply level of inter-regional education, which comprehensively expresses the balance and sufficiency of LES, as well as the relative initiative and competitiveness reflected therefrom.

According to the above literature, direct tax positively impacts local government livelihood expenditures, and PIT is a typical example of direct tax. Therefore, this study proposed the following hypothesis:

**H1:** The tax structure represented by the proportion of PIT positively impacts the relative power of LES.

Meanwhile, comparing and clarifying the impact mechanisms between Western and non-Western tax systems is crucial to ascertain the theoretical and practical implications of China’s system. The foot voting mechanism in Charles’ [27] model explains why European and American countries attach importance to the supply of local livelihood services; he argues that in the western regions dominated by direct taxes, such as PIT, attracting regional tax sources encourages population inflows, suggesting that livelihood services that satisfy residents are important. However, this mechanism remains unclear in China. First, the binding of China’s household registration and public service supply makes it difficult for the floating population to obtain public services equivalent to those of the registered population, as mentioned by Yang et al. [28]. Second, China’s tax system is based on indirect taxes, such as VAT; consequently, regional governments prefer to use productive investment, land concessions, and tax incentives to attract enterprises that can create VAT and business taxes. This study argues that although the proportion of PTI is not high for Chinese localities, the share of urban residents’ income that it reflects can drive the consumption rate of cities in an increasingly weak investment-led economy, which will have a multiplier effect on local economic development. Therefore, local governments will pay more attention to the status of consumer groups and the intensity of public service supply. To explore the impact mechanism between the tax structure and relative power of the LES, this study put forward the following hypothesis:

**H2:** Under the background of China’s dominant indirect tax, the tax structure represented by the proportion of PTI stimulates the relative power of the LES by increasing the local consumption rate and thereby enhancing the relative economic status of residents; however, it cannot significantly encourage local governments to attract floating populations and thus stimulate the relative power of LES.

In addition to taxation, local government debt is an important source of support for local governments’ fiscal expenditure. Since the 2008 global financial crisis, local government financing platforms have grown rapidly [29]. Especially with the sharp decline in local land income, governments are paying more attention to the use of debt policies [30]. In recent years, the risk of local government debt has become a major and urgent issue; however, there is no scholarly consensus on the impact of government debt on the supply of public goods for local livelihoods.

When studying the intergenerational cost of public services, Pedro [31] found that effective debt financing can balance the cost of public services between the current and next generation; that is, the effective debt scale expansion of local governments and the continuous increase in public investment are conducive to promoting the level of public services. Chen et al. [32] argues that government debt not only crowds out fiscal space due to the repayment of debt interest but also provides financial support for livelihood expenditures due to the income generated by its new debt; additionally, they also suggest that there is a positive relationship between government debt and public service supply. Jia et al. [33] concluded that the Chinese-style promotion and fiscal decentralization systems spawned competition among local officials with the goal of economic growth, which led to a blind-scale expansion of local government fiscal expenditure and excessive government debt impulses; moreover, they also advised that local governments will take on more debt for productive investments, which is not conducive to improving livelihoods and public services. Lan et al. [34] also reached a similar conclusion: fiscal reforms, such as tax and fee reductions, have slowed the growth of fiscal revenue and expanded the gap between fiscal revenue and expenditure, which caused local governments to increase the supply of public services through debt. However, based on economic rationality and the economic performance appraisals of promotion incentives, local governments are likely to prioritize their own interests and thus increase investment in productive projects for economic development and reduce investment in livelihood projects. In relevant researches on foreign countries, Cifuentes-Faura et al. [35] and Simionescu et al. [36, 37] believe that in order to reduce deficits and alleviate debt crises, the Spanish municipalities and Mexican states has had to control public expenditures such as local education.

Existing scholarly conclusions on the impact of local government debt on the supply of public goods are not consistent, and most focus on the supply level and ignore the relative power of LES. However, recognizing the vital role of LES, this study proposed the following hypothesis:

**H3:** The impact of local government debt on the relative power of LES may have non-linear effects

Simply studying the impact of government debt on the relative power of LES is insufficient. As an important part of the financial system, debt issuance must be considered systematically, which requires an in-depth exploration of the adjustment mechanism between government debt and education supply. Mao et al. [38] believe that safely resolving local government debt risks depends on their enhancing governance capabilities and improving governance efficiency. The research group "China ’s local government and financing platform debt analysis" [39] divides the resolution of local government debt risk into two stages: short-term and medium- and long-term. Short-term resolutions mainly focus on promoting the "control of increment and digestion of stock" of invisible debt, while medium- and long-term resolutions focus on deepening the mechanism reform and strengthening the construction of the local government’s governance capacity. Cifuentes-Faura et al. [40, 41] and Dzhumashev et al. [42] believe that it is impossible to provide effective government public services without improving taxation and management through increased transparency and efficiency in the audit process and effectively suppressed corruption. The improvement of local governance is also an important way to enhance the supply of public goods for people’s livelihood, which has been confirmed in Yang et al.’s [43] and Wang et al.’s [44] research from the perspective of environmental public goods.

The local government’s effective governance is an important factor in alleviating local debt risk and promoting local livelihoods, but ineffective local governance can suppress the relationship between local debt and the relative power of LES. Therefore, this study proposed the following hypothesis:

**H4:** The local government’s governance adjusts the relationship between local debt and the relative power of LES.

Prior literature discussing livelihood-oriented fiscal expenditures, such as education, focused on the absolute value of public expenditures or the absolute differences in expenditures between regions, with less attention paid to the relative initiative of the local government’s public expenditures. However, this object had to be assessed to address the insufficient and unbalanced supply of public goods in China. There are relatively few articles studying the impact of tax structure on LES. Instead, theoretical modeling analysis is the focus. Few empirical analysis studies have comprehensively explored the impact mechanism between the two. In China, where indirect taxes rather than direct taxes are predominant, research on direct taxes represented by PIT and their impact mechanisms is typical. Finally, some studies assessed the impact of government debt on local livelihood expenditures; however, literature exploring the adjusting mechanism of the government’s governance between these two factors is lacking. Therefore, this study will enrich previous literature in this regard.

## 3. Empirical strategy

### 3.1. Model settings

#### 3.1.1. Benchmark model setting

After successfully crossing the poverty line and meeting the basic indicators of livelihood security, various regions in China now face demand for the high-quality development of livelihood security. However, in light of the economic downturn and increasing financial pressure, it is more realistic and urgent to fully release the structural dividend of fiscal revenue and tap into the endogenous power of local governments’ livelihood service supply. This study used a fixed-effects model to test the impact of tax structure and government debt on the relative power of LES in prefecture-level cities. The empirical models were as follows:

$$y_{it}=\beta_{0}+\beta_{1}indi_{it}+\beta_{2}debr_{it}+\beta_{3}X_{it}+\mu_{i}+\sigma_{t}+\varepsilon_{it}$$ (1)

$$y_{it}=\beta_{0}+\beta_{1}indi_{it}+\beta_{2}debr_{it}+\beta_{3}debr_{it}I\left(debr_{it}<\omega_{1}\right)+\beta_{4}debr_{it}I\left(debr_{it}>=\omega_{1}\right)+\beta_{5}X_{it}+\mu_{i}+\sigma_{t}+\varepsilon_{it}$$ (2)

In the above equation, *t* represents time; *i* represents the city; *y*_*it*_ represents the relative power of LES in prefecture-level cities; *indi*_*it*_ represents the tax structure of prefecture-level cities, which is expressed as the proportion of PTI revenue to budgetary fiscal revenue; *debr*_*it*_ represents local debt, which is expressed as the proportion of urban investment debt to annual GDP; and ***I*(**·**)**is an indicator function, with a value of 1 when the corresponding condition is true, otherwise it is 0.

Most existing literature directly uses the proportion or scale of livelihood expenditure as an indicator of high-quality livelihood development or public services equity. However, these indicators spike rigidly upward with social and economic development and only represent the absolute—not relative—amount of local livelihood expenditures. Therefore, this study constructed the following indicators to reflect the relative power of LES. First, *y*_*it*_ reflects the level of high-quality LES development and highlights the endogenous power of LES from the perspective of balancing local education development and was calculated as follows:

$$y_{it}=\left(edu_{it}/\;expenditure_{it}\right)/\left(\sum_{i=1}^{n}edu_{it}/\sum_{i=1}^{n}\;expenditure_{it}\right)$$ (3)

*edu*_*it*_ and *expenditure*_*it*_ respectively represent the education and budgetary fiscal expenditures of prefecture-level city *i* in year *t*; *n*_*i*_ represents the number of cities in the province where city *i* is located; and $\sum_{i=1}^{n}edu_{it}$ and $\sum_{i=1}^{n}\;expenditure_{it}$ respectively represent the sum of the education and budgetary fiscal expenditures of all cities in this province in year *t*. This index can better reflect the relative power of LES from the perspective of inter-regional education equalization and development.

In Model 1 and Model 2, *X*_*it*_ is a series of control variables. Some represent the city’s economic situation, including per capita GDP, In(*pgdp*_*it*_); the ratio of total imports and exports to GDP, *open*_*it*_; and the total amount of new fixed assets (with 2010 as the base period, deflated according to the price index of fixed assets), In (*nfa*_*it*_). Some represent the city’s financial status, such as the fiscal self-sufficiency rate, (*fssr*_*it*_), and the proportion of the sum of VAT and business tax to the fiscal revenue in the city budget, *pvat*_*it*_. Last, some represent population characteristics, such as urbanization rate, (*urb*_*it*_), and population density, In (*den*_*it*_).

#### 3.1.2. Adjusting model settings

Even if the impact of government debt on the relative power of the LES is known, local governments still cannot stop using or indefinitely use local debt, and local government debt and local governance are inseparable. Therefore, the relationship between government debt and the relative power of the LES should be considered in adjusting local governance to derive more precise debt policies for the sustainable development of local finance, livelihood, and the economy. Therefore, the following model was constructed:

$$y_{it}=\beta_{0}+\beta_{1}indi_{it}+\beta_{2}debr_{it}+\beta_{3}X_{it}+\mu_{i}+\sigma_{t}+\varepsilon_{it}$$ (4)

$$y_{it}=\theta_{0}+\theta_{1}indi_{it}+\theta_{2}debr_{it}+\theta_{3}\;gove_{it}+\theta_{4}X_{it}+\mu_{i}+\sigma_{t}+\varepsilon_{it}$$ (5)

$$y_{it}=\gamma_{0}+\gamma_{1}\;indi_{it}+\gamma_{2}\;debr_{it}+\gamma_{3}\;gove_{it}+\gamma_{4}\;debr_{it}*\;gove_{it}+\gamma_{5}X_{it}+\mu_{i}+\sigma_{t}+\varepsilon_{it}$$ (6)

Before the adjustment regression analysis, this study centralized the independent, adjustment, and cross variables. The adjustment variable—local governance, *gove*_*it*_—is the ratio of the number of urban public administrations to GDP; the smaller the value, the higher the efficiency of local governance.

### 3.1.3 Intermediary mechanism

Tibout’s foot voting theory has been widely recognized in the context of fiscal decentralization in Western tax systems dominated by direct taxes. As stated above, local governments often need to attract floating populations sensitive to the local public services supply; hence, population inflow directly reflects the supply of local public services. However, given China’s special tax structure dominated by indirect taxes and the special household registration system, in which residents’ livelihood welfare such as education is bound to their registered residence rather than their own residence, it is of more special international comparative significance to explore the impact mechanism by which tax structure impacts the relative power of the LES,; more importantly, it is more practical for local governments under financial pressure to amplify the educational dividend of the tax system structure through intermediary mechanisms instead of increasing the tax burden.

The bootstrap method is the most widely used approach for directly testing the multiplication of coefficients [45]. Accordingly, adopting the proportion of the floating population (pm_it_) and urban consumption rate (cr_it_) in city i in year t as intermediary variables, this study employed the bootstrap method [46] to verify the intermediate mechanism between the tax structure and relative power of LES.

### 3.2. Data sources and descriptive statistics

Data were collected from 274 prefecture-level cities from 2010 to 2019. Data for the explained, adjustment, intermediary, and main control variable were taken from China Urban Statistical Yearbooks and city statistical bulletins city. Data on urban investment debt and PIT income for the core explanatory variables were taken from the WIND database, statistical yearbooks, and city statistical bulletins. Urban administrative division data were obtained from the China Administrative Division Network; to eliminate the impact of administrative division changes, urban samples with administrative division changes between 2010 and 2019 were deleted. Finally, for a very small number of missing values in the sample, average interpolation was used to fill in the data.

Table 2 presents the descriptive statistics. Tax structures and government debt significantly differed across regions. Considering the influence of outliers, we introduced a 1% tail shrinkage before and after all continuous variables. From the main variable trend from 2010 to 2019, the relative power of LES and tax structures showed an overall downward trend, which is related to the weak supply of local government livelihood services and the reduction and exemption of PIT; government debt first decreased and then increased.

**Table 2:** Descriptive statistics.

Variable Attribute	Variable	N	Mean	SD	Min	Max
Explained variables	y	2740	1.042	0.177	0.602	1.564
	y2	2740	1.057	0.228	0.534	1.638
Core explanatory variables	indi	2740	0.027	0.0158	0.008	0.0935
	debr	2740	0.209	0.294	0.0028	1.793
Moderating variable	gove	2740	0.0326	0.0184	0.0069	0.0976
Mediating variables	cr	2740	0.388	0.142	0.000029	1.509
	pm	2466	-0.00013	0.123	-3.508	0.773
Control variables	ln(den)	2740	5.751	0.894	2.893	7.301
	ln(pgdp)	2740	10.66	0.581	9.372	12.05
	open	2740	0.182	0.284	0.0021	1.613
	urb	2740	0.538	0.152	0.204	1
	fssr	2740	0.469	0.224	0.111	1.027
	pvat	2740	0.31	0.0842	0.122	0.554
	ln(nfa)	2740	18.18	0.823	16.35	20.23

Note: N = Number of observations, SD = Standard deviation.

## 4. Empirical results

### 4.1. Baseline regression results

Based on Model 1, we applied a two-way fixed regression effect method to verify the impact of the tax structure and government debt on the relative power of LES. Table 3 presents the results (Columns 1–3). Tax structure had a significant positive impact on the relative power of LES; an increase in the proportion of VAT in fiscal budget revenue increases the relative power of LES; these results are consistent with those of relevant literature [23, 24]. These may be because PIT and VAT stimulate taxpayers’ awareness of their rights and thus strengthen their expectations of LES management quality, which may enhance government motivation and responsibility for LES. Low PIT transferability and concealment increases tax pain and impacts the relative power of LES. From the perspective of linear effects, government debt had a significant positive impact on the relative power of LES, and from the perspective of nonlinear effects, the impact of government debt on the relative power of LES is initially negative and then positive. This is related to the priority of the use of local debts.

**Table 3:** Benchmark regression results.

	(1)	(2)	(3)	(4)
Variables	y	y	y	y
indi	1.16275***	1.11391***	1.23548***	1.16898***
	(0.29122)	(0.29193)	(0.25300)	(0.28984)
debr		0.03624**		0.03273*
		(0.01717)		(0.01701)
debr<0.4830			-0.06033**	
			(0.02930)	
debr> = 0.4830			0.03533**	
			(0.01710)	
pvat	0.24717***	0.24768***	0.23576***	0.24971***
	(0.03963)	(0.03960)	(0.03811)	(0.03934)
fssr	0.33290***	0.33423***	0.29692***	0.33444***
	(0.03111)	(0.03110)	(0.02767)	(0.03090)
ln(den)	0.02485	0.02423	0.00171	
	(0.04683)	(0.04680)	(0.04658)	
ln(pgdp)	-0.05763***	-0.06075***	-0.07250***	-0.06787***
	(0.01573)	(0.01579)	(0.01383)	(0.01429)
open	-0.05956***	-0.05943***	-0.05971***	-0.05996***
	(0.02205)	(0.02204)	(0.02178)	(0.02185)
urb	0.04366	0.04557	0.08447**	
	(0.04505)	(0.04503)	(0.04269)	
ln(nfa)	-0.01912	-0.02377	0.01228	
	(0.01612)	(0.01626)	(0.01313)	
constant	1.58415***	1.69802***	1.30450***	1.50548***
	(0.34773)	(0.35165)	(0.30664)	(0.14524)
observations	2,740	2,740	2,740	2,740
R-Squared	0.1145	0.1161	0.114	0.1149
Year	YES	YES	YES	YES
City	YES	YES	YES	YES
F-value	18.62	17.86	31.56	21.21
Number of id	274	274	274	274

Notes: The values in brackets are standard errors; ***, **, and * represent significance at the 1%, 5%, and 10% levels, respectively; “Yes” means that we have controlled for individual effects, time effects, and control variables.

After referring to relevant literature [32, 47, 48], this study further considered how control variables may affect the relative power of LES. It does not increase with an increase in per capita GDP or the degree of opening to the outside world but does increase significantly with increases in local financial self-sufficiency. Although some control variables were not significant, the estimation results were consistent after removing these variables (Column 4 of Table 3). However, to maintain the consistency of the results and facilitate comparative analysis, these control variables were included in the subsequent regression.

### 4.2. Robustness test

To ensure the reliability of the benchmark results, a robustness test was conducted from four aspects: eliminating some samples, replacing the core explanatory variables, replacing the explained variables, and changing the estimation method (Table 4). The first test eliminated some samples. Since provincial capitals and municipalities differ from prefecture-level cities in terms of political status, economic strength, and policy support, this test excludes samples of provincial capitals and municipalities from the regression; the results are consistent with the benchmark results (Column 1 of Table 4). The second test replaced the variables. The lagged one-period tax structure is the replacement variable. The substitution variables for the explained variables are shown in Eq (7). The empirical results are consistent with the benchmark regression results (Columns 2–3 of Table 4). The third test replaced the empirical model and applied the random-effects estimation method. The results are consistent with the benchmark regression (Column 4 of Table 4). The fourth test was an endogeneity test. The instrumental variable for tax structure was *eniv*_*it*_, with data from the investment index of the urban inclusive financial index jointly issued by Peking University and Alibaba. From the perspective of the correlation principle, the amount of PIT reflects resident income level, which directly affects resident enthusiasm for financial investment. The proportion of PIT and resident financial investment meet the correlation principle and pass the weak instrumental variable test. The results (Column 5 of Table 4) are consistent with the benchmark regression and pass the unrecognizable test.

**Table 4:** Robustness regression results.

	(1)	(2)	(3)	(4)	(5)
Variables	y	y	y2	y	y
indi	1.41282***		1.57205***	0.97480***	
	(0.26856)		(0.28547)	(0.23769)	
debr	0.05459***	0.04890***	0.06857***	0.01464	0.06949**
	(0.01876)	(0.01799)	(0.01933)	(0.01547)	(0.02713)
L.indi		0.46733*			
		(0.25227)			
ln(einv)					3.54577*
					(1.86262)
pvat	0.21891***	0.29330***	0.21865***	0.13922***	0.15652***
	(0.04146)	(0.03891)	(0.04316)	(0.03580)	(0.06067)
fssr	0.30590***	0.31072***	0.38956***	0.16302***	0.43512***
	(0.03050)	(0.02849)	(0.03132)	(0.02298)	(0.05154)
ln(den)	0.01731	0.04035	0.04505	0.00544	-0.02081
	(0.04944)	(0.04802)	(0.05258)	(0.01006)	(0.06847)
ln(pgdp)	-0.07569***	-0.05968***	-0.05332***	-0.08486***	-0.05024**
	(0.01475)	(0.01579)	(0.01567)	(0.01183)	(0.02326)
open	-0.05621**	-0.06414***	-0.04825*	-0.09432***	-0.0323
	(0.02271)	(0.02338)	(0.02463)	(0.01857)	(0.03356)
urb	0.07258	0.10078**	0.09173*	-0.04377	0.08569
	(0.04736)	(0.04330)	(0.04802)	(0.03780)	(0.06583)
ln(nfa)	0.00692	0.00605	-0.01408	0.00525	0.00639
	(0.01399)	(0.01372)	(0.01474)	(0.00968)	(0.01898)
constant	1.33139***	1.03191***	1.27432***	1.71180***	
	(0.33031)	(0.31663)	(0.34734)	(0.13450)	
observations	2,430	2,466	2,740	2,740	1,644
R-Squared	0.1058	0.0927	0.1071	0.0966	0.067
Number of id	243	274	274	274	274
F-value/Wald chi2-value	28.22	24.77	32.75	277.95	16.61
Underidentification test P value					0
Weak identification test F value					40.362

Notes: The values in brackets are standard errors; ***, **, and * represent significance at the 1%, 5%, and 10% levels, respectively.


$$y2_{it}=\left(edu_{it}/\;expenditure_{it}\right)/\left(\sum_{i=1}^{n}edu_{it}/\sum_{i=1}^{n}\;expenditure_{it}\right)$$ (7)

*edu*_*it*_ and *expenditure*_*it*_ respectively represent the annual education and budgetary fiscal expenditures of prefecture-level cities, *n* represents the number of cities in the sample, and $\sum_{i=1}^{n}edu_{it}$ and $\sum_{i=1}^{n}\;expenditure_{it}$ respectively represent the sum of the annual education and budgetary fiscal expenditures of all cities in the sample.

### 4.3. Mechanism testing

Since tax and debt policies comprise major regional and national fiscal policies, the positive impacts of the tax structure and government debt on the relative power of LES may not be infinitely amplified in reality. Therefore, this study explored the mechanism between them from the perspective of mediating and moderating effects.

This study used the bootstrap method to analyze the mediating effect between tax structure and the relative power of LES. Based on Charles’ [27] theory above, this study first verifies the mediating role of the proportion of floating population in the tax structure and the relative power of LES. The total effect of the tax structure on the relative power of LES is significantly positive; however, the mediating effect coefficient of the floating population is negative and insignificant (Table 5), which is similar to Yang et al.’s [49] conclusion. China’s prefecture-level cities have not fully followed the rules for matching the size of the floating population with the corresponding education supply, and there is still a significant lack of power in the education supply. Fiscal taxes in China are dominated by VAT and business tax for these years, the tax source of which is mainly from enterprises rather than residents. In addition, residents’ livelihood welfare, such as education, is bound to registered residence rather than actual residence. The above two encourage the governments’ prioritization of productive investment over livelihood supports.

**Table 5:** Mediation effect analysis results.

Explained variables variable	Mediating variable	Number of reps	Effect analysis	Coeffi- cient	Standard deviation	95% Confidence interval	
y	pm	1000	ind_effect	-0.0327	0.0384	-0.108	0.0427
			dir_effect	1.1721	0.2805	0.6223	1.7218
		2000	ind_effect	-0.0327	0.0382	-0.1074	0.0421
			dir_effect	1.1721	0.2875	0.6085	1.7356
	cr	1000	ind_effect	0.2238	0.0697	0.0873	0.3604
			dir_effect	1.0676	0.2797	0.5193	1.6159
		2000	ind_effect	0.2238	0.0675	0.0915	0.3561
			dir_effect	1.0676	0.2754	0.5279	1.6074

Notes: Number of reps represents the number of random repeated sampling with return; dir_ Effect represents the direct effect of independent variable on dependent variable after controlling intermediary effect; Ind_Effect represents the intermediary effect; The 95% confidence interval excluding 0 is the basis for determining the significance of indirect and direct effects.

Furthermore, this study verified the mediating role of the consumption rate. The mediating effect coefficient of the consumption rate is significantly positive and accounts for nearly 25% of the total effect (Table 5), proving that tax structure can enhance the power of LES by increasing the consumption rate; the higher the proportion of PIT, the more high-income people consume, which promotes local consumption, directly drives local economic development, provides financial support for LES, and motivates local governments to pay more attention to the educational and livelihood needs of consumer groups.

Verifying and comparing the two intermediary channels suggested that the low proportion of PIT does not simulate the relative power of LES by encouraging local governments to attract floating populations, but instead by enhancing residents’ relative economic status through increasing the local consumption rate.

Based on Eqs (4)–(6), this study examined the moderating effect of local governance on the relationship between government debt and the relative power of LES; the estimated results are shown in Table 6 (Columns 1–3). The influence coefficient of local governance is positive, indicating that more investment in local governance can improve the relative power of LES. Meanwhile, local governance investment has a significant negative adjustment effect on the relationship between government debt and the relative power of LES; therefore, the higher the local governance investment, the smaller the promotion effect of government debt on the relative power of LES. Although increasing investment in local governance is conducive to LES, the local government should remain attentive to the counter effect of excessive governance investment and strive to improve local governance efficiency to better apply government debt issuance income to areas with major shortages, in step with the concept of the government’s living on tight budget and the people’s living in good days.

**Table 6:** Moderating effect analysis results.

	(1)	(2)	(3)
Variables	y	y	y
indi	1.11391***	1.11148***	0.96858***
	(0.29193)	(0.29182)	(0.29414)
debr	0.03624**	0.03500**	-0.00003
	(0.01717)	(0.01718)	(0.01995)
gove		0.70617*	0.20747
		(0.41307)	(0.43707)
debr*gove			-3.21172***
			(0.01459)
constant	1.69802***	1.40906***	1.47365***
	(0.35165)	(0.39004)	(0.38424)
observations	2,740	2,740	2,740
R-Squared	0.1161	0.1171	0.1213
Year	YES	YES	YES
City	YES	YES	YES
F-value	17.86	17.08	16.89
Number of id	274	274	274

**Notes**: The values in brackets are standard errors; ***, **, and * represent significance at the 1%, 5%, and 10% levels, respectively; “Yes” means that we have controlled for individual effects, time effects, and control variables.

### 4.4. Heterogeneity test

China’s diverse landscapes, economics, and systems may cause different cities to align with different research conclusions. Historically, studies of regional heterogeneity in China divided regions into eastern, central, western, and northeastern regions to reflect the differences in economic and social development brought about by heterogeneous regional policies. Recently, the continuous advancement of balanced regional development strategies has drawn attention to regional differences between the north and south, which reflect the development gap resulting from their own market-oriented systems. Based on the above two regional division methods, this study examined regional differences in the impact of the tax structure and government debt on the relative power of LES.

First, based on the division of the eastern, central, western, and northeastern regions by the National Bureau of Statistics, we merged the western and northeastern regions into the western region. Tax structure has a significant positive impact on the relative power of LES in the eastern and western regions, but this effect is not significant in the central region (Table 7, Columns 1–3). Government debt has a significant positive impact on the relative power of LES in the eastern and western regions, but a significant negative impact in the central region. Increasing government debt in the central region may support the expansion of productive investment and thus compress the supply of public goods for livelihood, such as education.

**Table 7:** Heterogeneity analysis.

	Eastern region	Central region	Western Regions	Northern region	Southern region
Variables	y	y	y	y	y
indi	1.29366**	0.82552	1.73615***	0.98863**	1.10846***
	(0.51013)	(0.53749)	(0.61909)	(0.50292)	(0.36348)
debr	0.04059	-0.06462*	0.07364**	0.05096*	0.02039
	(0.02584)	(0.03668)	(0.03091)	(0.03065)	(0.02079)
pvat	0.17964**	0.24164***	0.21683***	0.29495***	0.19938***
	(0.07737)	(0.07689)	(0.06967)	(0.05868)	(0.05514)
fssr	0.36786***	0.34544***	0.36484***	0.41383***	0.31529***
	(0.06240)	(0.04695)	(0.07474)	(0.06026)	(0.03634)
ln(den)	-0.02445	-0.25005***	0.13374	0.20398***	-0.15650**
	(0.06963)	(0.09349)	(0.09314)	(0.07231)	(0.06212)
ln(pgdp)	-0.01337	-0.12011***	-0.08493***	-0.09096***	-0.04651*
	(0.02939)	(0.03850)	(0.03100)	(0.02627)	(0.02481)
open	-0.02773	-0.17853**	-0.10368**	-0.03861	-0.07513**
	(0.03299)	(0.08442)	(0.04605)	(0.03299)	(0.03029)
urb	-0.14755*	-0.00934	0.18774*	0.13213**	-0.13118
	(0.08307)	(0.11180)	(0.09744)	(0.05682)	(0.08738)
ln(nfa)	-0.03605	-0.03686	-0.08414**	-0.06098**	-0.02613
	(0.02578)	(0.04744)	(0.03484)	(0.02868)	(0.02082)
constant	1.75694**	4.21392***	2.43580***	1.62191***	2.78599***
	(0.68508)	(1.03441)	(0.75758)	(0.52699)	(0.55873)
observations	840	770	790	1,230	1,510
Number of id	84	77	79	123	151
R-Squared	0.157	0.1735	0.1347	0.1347	0.1264
F-value	7.63	7.87	6	9.42	10.78
Year	YES	YES	YES	YES	YES
City	YES	YES	YES	YES	YES

**Notes**: The values in brackets are standard errors; ***, **, and * represent significance at the 1%, 5%, and 10% levels, respectively; “Yes” means that we have controlled for individual effects, time effects, and control variables.

Second, based on Sheng et al.’s [50] division standard, the sample was divided into northern and southern regions. The results reveal that tax structure significantly enhances the relative power of LES in both regions and that this effect is deeper in the southern region (Table 7, Columns 4–5). This may be related to the southern region’s more mature market-oriented system and better government service awareness. Meanwhile, increasing government debt enhances the relative power of LES in the northern region, but the effect is not obvious in the southern region. This may be due to the southern region’s better economic development and fiscal revenue levels, which reduce the dependence of education supply expenditures on government debt.

## 5. Policy suggestion

These findings have the following policy implications. First, given increasing fiscal pressure and the government’s urgent need to expand the supply of public goods for both production and livelihood, policymakers should pay more attention to the key role of the tax structure. Specifically, to improve the relative power of LES, policymakers should firmly increase the proportion of direct taxes, improve the reform of the PIT including abolishing the tax-free treatment of personal capital gains, expanding the scope of personal comprehensive income and fairly taxing it with personal labor income, lowering the maximum marginal tax rate of personal comprehensive income, setting industry differentiated tax rates, and strict tax collection and management, and adjust the proportions of the central and local divisions of PIT in step with the growing local expenditure obligations.

Second, the growth of urban consumption rate and population inflow rate should be emphasized to stimulate the relative power of LES. Hence, the local government should further reform the household registration system and improve basic public services according to the regional permanent residents instead of registered population.

Finally, policymakers should consider the joint effects of local governance and debt on the relative power of LES. Although local debt can alleviate the financial constraints on the supply of livelihood services such as education, the local government should scientifically control the scale of government debt,reasonably use education special bond to ensure local education. Meanwhile, They should work to improve the efficiency of local governance including optimally reforming government budget accounting, strengthening the disclosure of financial information, reconstructing the mechanism of government supervision and accountability, enhancing citizens’ right to speak in financial decision-making.

## 6. Conclusion

Education is related to fundamental plans for sustainable regional and national development. This study explored the impact of fiscal structure and governance debts on the relative power of LES to uncover insights that are useful for balancing local finance, strengthening the education supply, and driving sustainable economic and social development. The results revealed that the relative power of LES ultimately increases with the increases in tax structure—through the tax structure’s positive impact on local consumption—and government debt—based on the level of investment in local governance. Notably, influence of the tax structure is strongest in the southern region and influence of government debt is strongest in the north, which reflects that in the southern regions where the market economy system is more developed, the relative power of LES has a greater response to the tax system structure and a smaller dependence on local government debt.

There are three novelties in this study. First, it proposes the concept of the relative power of Local education Supply and constructs the relative power index of LES, which highlight the proactive characteristics of LES by balancing its inter-regional gaps. And it directly points out the latest contradiction of imbalanced and insufficient supply of basic public services such as education in China at present and focus on balancing regional disparities and transitioning to active competitive supply rather than passive index supply, which are the previous studies fail to reflect. Second, based on a fiscal background different from most Western countries, in which the fiscal revenue of Chinese local governments is dominated by indirect taxes rather than direct taxes and many local urban investment bonds. This study clarifies the influence of the local tax structure and government debt on the relative power of Local education Supply from theoretical and empirical perspectives. On one hand, existing literature pays little attention to tax structures—an important part of the financial system, so an in-depth analysis of fiscal structure can yield more systematic and comprehensive financial theory that is applicable to Local education Supply. One the other hand, focusing on the special fiscal background of local governments in China, this study provide a theoretical and practical sample for the balanced and sustainable development of global education. Finally, the study uncovers the intermediary and adjustment mechanisms of the local tax structure and government debt affecting the relative power of the LES and provides policy implications, and based on China’s special registered residence system, by comparing two different intermediary mechanisms, the study yields an international comparison and policy reference.

However, owing to limitations in data and research conditions, there are still some gaps that require further exploration. First, owing to data limitations, the government debt data selected in this study is the local urban investment debt data, which lacks examination of special education expenditure debt. Otherwise, it is possible to more accurately and meticulously observe the impact of local debt on the relative power of LES and propose corresponding policy recommendations. Second, the findings of this study indicate that it is necessary for local governments to carry out tax system reform and attach importance to the increase of the proportion of PIT, thus stimulating the endogenous power of LES. However, owing to the lack of data, analysis has not been conducted from other perspectives, such as how to effectively enhance the relative power of LES in areas where the degree of tax reform is not considerable and the proportion of PIT is relatively low. The above issues require further discussion in the future.

## Supporting information

S1 Table The data of the variables involved (except for the variable *debr*). (XLSX)
